# Antifungal Activity of Extracts and Prenylated Coumarins Isolated from *Baccharis darwinii* Hook & Arn. (Asteraceae)

**DOI:** 10.3390/molecules15074898

**Published:** 2010-07-13

**Authors:** Rita R. Kurdelas, Beatriz Lima, Alejandro Tapia, Gabriela Egly Feresin, Manuel Gonzalez Sierra, María Victoria Rodríguez, Susana Zacchino, Ricardo D. Enriz, Monica L. Freile

**Affiliations:** 1 Laboratorio de Productos Naturales Patagónicos (LAPRONAP), Facultad de Ciencias Naturales, Universidad Nacional de la Patagonia San Juan Bosco, Km 4, CP 9000, Comodoro Rivadavia, Chubut, Argentina; E-Mail: rkurdelas@unpata.edu.ar (R.R.K.); 2 Instituto de Biotecnología - Instituto de Ciencias Básicas, Universidad Nacional de San Juan, Av. Libertador General San Martín 1109 (O), CP5400, San Juan, Argentina; E-Mails: atapia@unsj.edu.ar (A.T.); blima@unsj.edu.ar (B.L.); gferesin@unsj.edu.ar (G.E.F.); 3 Facultad de Ciencias Bioquímicas y Farmacéuticas, Farmacognosia y Biología Vegetal. Universidad Nacional de Rosario, Suipacha 531, CP2000, Rosario, Argentina; E-Mail: szaabgil@citynet.net.ar (S.Z.); 4 Facultad de Química, Bioquímica y Farmacia. Universidad Nacional de San Luis, Chacabuco 917, CP5700, San Luis, Argentina; E-Mail: denriz@unsl.edu.ar (R.D.E.)

**Keywords:** *Baccharis darwinii*, Asteraceae, prenylated coumarins, auraptene, antimicrobial activity

## Abstract

The petroleum ether extract of *Baccharis darwinii* showed activity against *Cryptococcus* *neoformans* and dermatophytes. Bioactivity-guided fractionation of *Baccharis darwinii* has resulted in the isolation of three coumarins: 5’-hydroxy aurapten (anisocoumarin H, **1**), aurapten (7-geranyloxycoumarin, **2**) and 5’-oxoaurapten (diversinin, **3**). The structures of these compounds were characterized by spectroscopic methods. These compounds were evaluated for their antimicrobial activity against a panel of each, bacteria and fungi. Compound **3** showed the best activities against *Microsporum gypseum*, *Trichophyton rubrum* and *Trichophyton mentagrophytes* with MICs = 15.6 µg/mL, followed by compound **1** whose MICs against the same fungi were 62.5 µg/mL. In addition they showed fungicidal rather than fungistatic activity. Both compounds showed moderate activity (MICs = 125 µg/mL) against *Cryptococcus neoformans*. This is the first report of the presence of compound **1** in *B. darwinii*.

## 1. Introduction

Currently, medicinal plants are widely used as home remedies or as alternative treatments by both rural and urban inhabitants in developing countries [1], which can be explained in part by the high cost of industrialized medicine. In turn, many extracts from plants have shown biological activities of interest, which justifies the search of potentially active compounds in them. 

A matter of concern in public health in the last decades is the increasing resistance of bacteria and fungus to antimicrobial drugs and the growing number of immunocompromised patients undergoing fatal fungal infections, since the available antifungals are scarce, sometimes ineffective and not always safe. 

The genus *Baccharis* L. possesses the highest number of species (between 400 and 500) within the Asteraceae family [2]. It was found only in American countries from the south of United States to the austral end of Argentina and Chile, occupying a great variety of environments. In the Argentine Republic, it is represented by 96 species [2], and among them, *Baccharis darwinii*, commonly known as “chilca”, can be found in the far south of South America that is, in the arid Patagonia region of Argentina.

Several species of *Baccharis* genus are used in traditional medicine [3,4] for different ailments. Some species, known as “carquejas” (*B. trimera, B. crispa*, etc.) are used against hepatic disorders. Another species, known as “mio-mio” (*B. coridifolia*) is poisonous for livestock [5], while another, “mata-trigo” (*B. gilliesii*), is an invader of cultures. 

Studies on the biological activities of species of this genus have shown antimicrobial, antioxidant, anti-inflammatory and antifeedant activities [6,7,8,9,10,11,12,13,14,15,16,17]. Regarding the chemical constituents found in the genus, flavonoids and terpenoids are the most frequently reported [17,18,19,20]. Regarding *B. darwinii,* the presence of β-farnesene, aurapten, 5’-oxoaurapten, and 6’,7’-epoxy- and 6’,7’-dihydroxyaurapten were reported by Zdero *et al*. in a sample collected in the Argentinean Neuquén province [21]. Nevertheless, to the best of our knowledge there are no reports on the biological activities of *Baccharis darwinii*. We report here the antimicrobial activity of crude extracts of this plant collected in Chubut province, a southernmost province of Argentina, and the bioassay-guided fractionation of its most active extract which led to the isolation of the main compounds responsible for the activity. 

## 2. Results and Discussion

In the first step of our work, we evaluated the antimicrobial (antibacterial and antifungal) activity of the petroleum ether (PE), dichloromethane (DCM) and methanol (MeOH) extracts from aerial parts of *Baccharis darwinii*. Regarding antibacterial activity of the different extracts, MeOH did not show any activity (MIC > 1,000 µg/mL), PE showed only marginal activity (MIC ≥ 1,000 µg/mL) and DCM extract showed a very low activity against only two bacteria (MIC = 750 µg/mL) and marginal or null activity against the rest of bacteria of the panel.

In contrast, the three extracts showed better antifungal activities, mainly against dermatophytes and, in addition, they were shown to possess not only fungistatic but also fungicidal capacity (Table 1). Nevertheless, some differences could be observed among the activity of the three extracts (Table 1): the PE and MeOH extracts were the most active extracts mainly against *C. neoformans*. Between them, PE extract showed four-fold higher potency than MeOH extract against the same fungi. Among the different extracts, PE is of great interest since it showed a selective and good activity against *C. neoformans* and dermatophytes. On the basis of these results, PE was submitted to fractionation guided by antifungal activity as described in the Experimental section. 

**Figure 1:**
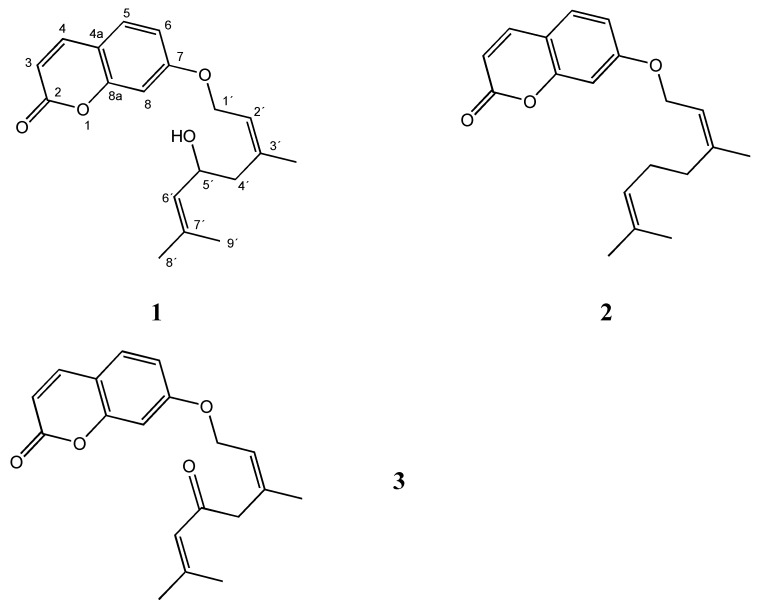
Structures of compounds **1-3**.

The nine sub-fractions obtained from PE extract (I-IX) were evaluated for antifungal properties against the same panel used for the crude extracts. Results (Table 2) showed that the best activity was displayed by sub-fraction VI against dermatophytes (MIC and MFC = 62.5 µg/mL). From this sub-fraction for successive fractionations, three known coumarins: 5’-hydroxyaurapten (anisocoumarin H, **1**), aurapten (7-geranyloxycoumarin, **2**) and 5’-oxoaurapten (diversinin, **3**) were isolated and their antifungal activities were evaluated. Of these, **1** has not been reported previously in *B. darwinii* while **2** and **3** have been reported previously in this plant [21]. The compounds were identified by comparison of their NMR data with literature vales (see Experimental). The corresponding structures are shown in Figure 1.

It is interesting to note that two of these coumarins, **1** and **3**, displayed antifungal activity against *C. neoformans* and dermatophytes (Table 2) and therefore both compounds would be the responsible for the antifungal activity observed in the PE extract of *B. darwinii*. Compound **3** possess a high fungistatic activity against the three dermatophytes tested, with MICs = 15.6 µg/mL and a fungicide capacity. This result is very interesting since *T. rubrum* and *T. mentagrophytes* are responsible for approximately 80–93% of chronic and recurrent dermatophytes infections in human beings. They are the ethiological agent of tinea unguium (producer of invasive nail infections), tinea manuum (palmar and interdigital areas of the hand infections) and tinea pedis (athlete’s foot), the last one being the most prevalent fungal infection in developed countries, and the first one accounting for 50% and 90% of all fingernail and toenail infections respectively [22]. 

In turn, although the activity displayed by **1** and **3** against *C. neoformans* (MIC = 125 µg/mL) is moderate, it is worthy to take into account that *C. neoformans* remains an important life-threatening complication for immunocompromised hosts, particularly for patients who have undergone transplantation of solid organs and, therefore, new compounds acting against this fungus are highly welcome [23]. The antimicrobial activity is considered very interesting in the case of MICs < 100 µg mL^-1^ for extracts and 10 µg mL^-1^ for isolated compounds [24]. Another interesting contribution of this work is that, since the three coumarins have been previously isolated from other species, such as anisocoumarin H (**1**) in *Clausena anisata* [25] and *Notopterygium forbesii* [26]; auraptene (**2**) in *Ferulago capillaris* [27], *Paliurus ramosissimus* [28]; diversinin (**3**) in *Ferula diversivittata* [29], this work encourages the beginning of antifungal studies of these species in order to evaluate their antifungal potentiality. 

## 3. Experimental

### 3.1. General

IR spectra were recorded in a FTIR Shimadzu Prestige-21 instrument; 1D and 2D NMR spectra were recorded using a Bruker Avance 300 in deuterated chloroform solutions, using standard Bruker software. GC-MS were run in a Perkin Elmer Autosystem XL Gas Chromatograph coupled to a Turbo Mass Spectrometer. Column: SE-30, 25 m × 0.22 mm ID, Scientific Glass Engineering (Ringwood, Victoria, Australia). 

### 3.2. Plant material

Aerial parts of *Baccharis darwinii* were collected at Comodoro Rivadavia, Chubut Province, Argentina at 45º 48’ 34.5” Latitude (S) and 67º 26’ 44” Longitude (O) in October 2007. The plant was identified by Prof. María Elena Arce, FCN, UNPSJB. A voucher specimen was deposited at Herbario Regional Patagónico (HRP 6154), Comodoro Rivadavia, Chubut, Argentina.

### 3.3. Extraction

The dried powdered aerial parts (450 g) were extracted successively with petroleum ether (PE), dichloromethane (DCM) and methanol (MeOH) at room temperature (3 × 1,000 mL, for 96 h each time). After evaporation to dryness under reduced pressure, PE, DCM and MeOH extracts were obtained in yields (w/w in terms of dry starting material) of 3.46%, 2.26% and 14,14%, respectively. 

### 3.4. Isolation

A representative sample from PE bioactive extract (13.13 g) was applied to a Sephadex LH-20 column (column length 37 cm, diameter 4 cm) equilibrated with: PE:MeOH:CHCl_3_ (2:1:1). After TLC comparison (silica gel, EtOAc:PE 2:8, as the mobile phase; detection under UV light followed by spraying with *p*-anisaldehyde), nine fractions were obtained: I: (0.2076 g), II: 1.1049 g, III: 1.2438 g, IV: 2.6301 g, V: 0.9964 g, VI: 3.6209 g, VII: 0.4913 g, VIII: 0.0180 g, IX: 0.0936 g.

#### 3.4.1. Pooled *fraction VI*

Fraction VI (3 g) was applied to a Sephadex LH-20 column (column length 37 cm, diameter 4 cm) equilibrated with: PE:MeOH:CHCl_3_ (2:1:1). After TLC comparison (silica gel, EtOAc:PE 2:8 as the mobile phase; detection under UV light followed by spraying with *p*-anisaldehyde), fractions were combined affording fifteen new fraction: VI_1_: 0.0022 g, VI_2_: 0.0034 g, VI_3_: 0.0113 g, VI_4_: 0.0209 g, VI_5_: 0.0436 g, VI_6_: 0.1155 g, VI_7_: 0.2904 g, VI_8_: 0.4154 g, VI_9_: 0.4587 g, VI_10_: 0.4793 g, VI_11_: 0.4424 g, VI_12_: 0.3260 g, VI_13_: 0.1543 g, VI_14_: 0.1015 g, VI_15_: 0.091g. From fractions VI_12_-VI_15_ pure compound **1** (91.6 mg) was obtained.

The potent bioactive antifungal fractions VI_7_– VI_11_ (749.2 mg) were applied onto a medium pressure chromatography column (MPCC, column length 60 cm, diameter 2 cm) containing 75 g silica gel (0.063-0.2 mesh, Merck 60) and eluted with 150 mL of cyclohexane-EtOAc 100:100 v/v. Some 350 fractions of 10 mL each were obtained. From fractions 44-64 pure compound **2** (72.6 mg) was obtained whereas from fractions 150-158 yielded mainly compound **3** (100.9 mg).

### 3.5. Isolated compounds

*7-(5-Hydroxy-3,7-dimethylocta-2,6-dienyloxy)-chromen-2-one* (*5’-hydroxy aurapten*, **1**). The NMR data were in agreement with those reported by Ngadjui *et al*. [25].

*7-(3,7-Dimethylocta-2,6-dienyloxy)-chromen-2-one* (*aurapten*, **2**). The NMR data were in agreement with those reported by Zdero *et al*. [21].

*7-(3,7-Dimethyl-5-oxoocta-2,6-dienyloxy)-chromen-2-one* (*5’-oxoaurapten*, **3**). The NMR data were in agreement with those reported by Zdero *et al*. [21].

### 3.6. Antimicrobial activity

#### 3.6.1. Microorganisms and media

For the antibacterial evaluation, strains from the American Type Culture Collection (ATCC) Rockville MD (USA), Malbrán Institute (MI), Pasteur Institute (PI) and from the Laboratorio de Microbiología (LM, Facultad de Ciencias Médicas, Universidad Nacional de Cuyo, Mendoza, Argentina) were used: *Escherichia coli* ATCC 25922, LM_1_-*Escherichia coli*, LM_2_-*Escherichia coli, Pseudomonas aeruginosa* ATCC 27853, LM-*Salmonella* sp., MI-*Salmonella enteritidis,* PI-*Yersinia enterocolítica*, *Staphylococcus aureus* methicillin-sensitive ATCC 29213 and *Staphylococcus aureus* methicillin-resistant ATCC 43300. Bacteria were grown on Müeller–Hinton agar medium.

For the antifungal evaluation, strains from the American Type Culture Collection (ATCC), Rockville, MD, USA and CEREMIC (C), Centro de Referencia en Micología, Facultad de Ciencias Bioquímicas y Farmacéuticas, Suipacha 531, 2000 Rosario, Argentina, were used: *Candida albicans* ATCC10231, *Candida tropicalis* C131, *Saccharomyces cerevisiae* ATCC9763, *Cryptococcus neoformans* ATCC32264, *Aspergillus flavus* ATCC9170, *Aspergillus fumigatus* ATTC26934, *Aspergillus niger* ATCC9029, *Trichophyton rubrum* C110, *Trichophyton mentagrophytes* ATCC9972 and *Microsporum gypseum* C115. Strains were grown on Sabouraud-chloramphenicol agar slants for 48 h at 30 ºC, maintained on slopes of Sabouraud-dextrose agar (SDA, Oxoid) and sub-cultured every 15 days to prevent pleomorphic transformations. Inocula were obtained according to reported procedures [30] and adjusted to 1–5 × 10^3^ cells/spores with colony forming units (CFU) /mL 

#### 3.6.2. Antibacterial susceptibility testing

Cultures less than 30 h old were touched with a loop and transferred to sterile Mueller-Hinton broth. The broth was incubated at 37 ºC until the growth reaches turbidity equal to, or greater than that of 0.5 Mc Farland standards. The culture was adjusted with sterile physiological solution to give final organism density of 5 × 10^5^ CFU/mL [31,32]. The antibacterial activity was evaluated with the agar dilution method using Mueller Hinton agar medium for Gram (+) and Gram (-) bacteria. Stock solutions of the compounds/extracts in DMSO were diluted to give serial two-fold dilutions that were added to each medium resulting in concentrations ranging from 10 to 1,000 µg/mL. The final concentration of DMSO in the assay did not exceed 1%. The antimicrobial agent cefotaxime (Argentia Pharmaceutica) was included in the assays as positive control. The plates were incubated for 24 h at 37 ºC. Minimum Inhibitory Concentration (MIC) was defined as the lowest compounds/extracts concentration showing no visible bacterial growth after incubation time. Tests were carried out in triplicate. Extracts and compounds with MICs ≤ 250 µg/mL were considered of interest. 

#### 3.6.3. Antifungal susceptibility testing

Minimum Inhibitory Concentration (MIC) of each extract or compound was determined by using broth microdilution techniques according to the guidelines of the Clinical and Laboratory Standards Institute (CLSI, formerly National Committee for Clinical and Laboratory Standards, NCCLS) for yeasts (M27-A2) and for filamentous fungi (M 38 A), [30]. MIC values were determined in RPMI-1640 (Sigma, St Louis, MO, USA) buffered to pH 7.0 with MOPS. Microtiter trays were incubated at 35 ºC for yeasts and hialohyphomycetes and at 28–30 ºC for dermatophyte strains in a moist, dark chamber. MICs were visually recorded at 48 h for yeasts, and at a time according to the control fungus growth, for the rest of fungi. Extracts with MICs ≤ 1,000 µg/mL and sub-extracts and compounds with MICs ≤ 250 µg/mL were considered active.

For the assay, stock solutions of pure compounds were two-fold diluted with RPMI from 1,000 or 250 µg/mL to 0.98 (final volume = 100 µL) and a final DMSO concentration ≤ 1%. A volume of 100 µL of inoculum suspension was added to each well with the exception of the sterility control where sterile water was added to the well instead. Ketoconazole (Sigma Chem. Co.), terbinafine (Novartis, Argentina) and amphotericin B (Sigma Chem. Co.), were used as positive controls. Endpoints were defined as the lowest concentration of drug resulting in total inhibition (MIC_100_) of visual growth compared to the growth in the control wells containing no antifungal. 

The Minimum Fungicidal Concentration (MFC) of each extract or compound was determined as follows: After determining the MIC, an aliquot of 5 µL sample was withdrawn from each clear well of the microtiter tray and plated onto a 150-mm RPMI-1640 agar plate buffered with MOPS (Remel, Lenexa, Kans.). Inoculated plates were incubated at 30 ºC, and MFCs were recorded after 48 h. The MFC was defined as the lowest concentration of each compound that resulted in total inhibition of visible growth in these plates.

## 4. Conclusions

Phytochemical study of *B. darwinii* extracts, bioguided by the antifungal assay, led to the isolation of the known prenylated coumarins 5’-hydroxyaurapten (**1**), aurapten (**2**) and 5’-oxoaurapten (**3**). This is the first report of the presence of compound **1** in *B. darwinii*. Among the three isolated compounds compound **3** showed the best activities against *M. gypseum*, *T. rubrum* and *T. mentagrophytes* with MICs = 15.6 µg/mL, followed by compound **1** whose MICs against the same fungi were 62.5 µg/mL. In addition their activity was shown to be fungicidal rather than fungistatic. Both compounds showed moderate activity (MICs = 125 µg/mL) against *C. neoformans*. 

## Figures and Tables

**Table 1:** Antifungal and antibacterial activity to *Baccharis darwinii* extracts (MIC/MFC, µg/mL).

Microorganisms	Extracts			Reference drugs		
**Fungus**	PE	DCM	MeOH	Amp B	Ket	Terb
*Candida albicans* ATCC 10231	>1,000	>1,000	>1,000	1	0.5	-
*Candida tropicalis* C 131 2000	>1,000	>1,000	>1,000	0.5	0.125	-
*Saccharomyces cerevisiae* ATCC 9763	>1,000	>1,000	>1,000	0.5	0.5	-
*Cryptococcus neoformans* ATCC 32264	125/250	>1,000	500/1,000	0.25	0.25	-
*Aspergillus flavus* ATCC 9170	>1,000	>1,000	>1,000	0.5	0.125	-
*Aspergillus fumigatus* ATCC 26934	>1,000	>1,000	>1,000	0.5	0.25	-
*Aspergillus niger* ATCC 9029	>1,000	>1,000	>1,000	0.5	0.5	
*Microsporum gypseum* C 115 2000	125/125	1,000/1,000	500/500	0.125	0.05	0.04
*Trichophyton rubrum* C 113 2000	62.5/125	500/1,000	250/1,000	0.075	0.025	0.025
*Trichophyton mentagrophytes* ATCC 9972	62.5/125	500/1,000	250/500	0.075	0.025	0.04

(*) Reference substances: Amp B: Amphotericin B, Ket: Ketoconazole, Terb: Terbinafine.

**Table 2:** *In vitro* evaluation of the antifungal activity of different fractions resulted from Sephadex percolation of PE extracts and compounds **1-3** from *B. darwinii* (MIC/MFC values are given in μg/mL).

Seph PE Fractions	*Ca*	*Ct*	*Sc*	*Cn*	*Mg*	*Tr*	*Tm*
Seph PE I-III	> 250	> 250	> 250	> 250	> 250	> 250	> 250
Seph PE IV	> 250	> 250	> 250	> 250	250/250	250/250	250/250
Seph PE V	> 250	> 250	> 250	250/>250	62.5/62.5	62.5/125	62.5/125
Seph PE VI	> 250	> 250	> 250	250/> 250	62.5/62.5	62.5/62.5	62.5/62.5
Seph PE VII	> 250	> 250	> 250	250/> 250	125/250	125/250	125/250
Seph PE VIII	> 250	> 250	> 250	250/>250	250 / 250	250/>250	250/>250
Seph PE IX	> 250	> 250	> 250	>250	> 250	>250	>250
**Compounds**							
**1**	> 250	> 250	> 250	125	62.5/125	62.5/62.5	62.5/62.5
**2**	> 250	> 250	> 250	> 250	> 250	> 250	> 250
**3**	> 250	> 250	> 250	125	15.6/125	15.6/125	15.6/125
**Reference drugs**							
Amphotericin B	0.25	0.25	1	0.50	0.12	0.07	0.07
Ketoconazole	0.25	0.25	0.50	0.50	0.04	0.02	0.02
Terbinafine					0.04	0.01	0.04

*Ca: Candida albicans* ATCC 10231; *Ct: Candida tropicalis* C 131; *Sc: Saccharomyces cerevisiae* ATCC 9763; *Cn: Cryptococcus neoformans* ATCC 32264; *Mg: Microsporum gypseum* C 115; *Tr: Trichophyton rubrum* C113; *Tm: Trichophyton mentagrophytes* ATCC 9972.
